# Emulsified isoflurane anesthesia decreases brain-derived neurotrophic factor expression and induces cognitive dysfunction in adult rats

**DOI:** 10.3892/etm.2014.1769

**Published:** 2014-06-10

**Authors:** FAN ZHANG, ZHAO-QIONG ZHU, DE-XING LIU, CHAO ZHANG, QI-HAI GONG, YU-HANG ZHU

**Affiliations:** 1 Department of Anesthesiology, Affiliated Hospital of Zunyi Medical College, Zunyi, Guizhou 563000, P.R. China; 2 Department of Pharmacology, Zunyi Medical College, Zunyi, Guizhou 563000, P.R. China

**Keywords:** emulsified isoflurane, cognitive dysfunction, plasma corticosterone, brain-derived neutrophic factor, nerve growth factor

## Abstract

Post-operative cognitive dysfunction (POCD) is a severe complication characterized by cognitive decline in patients following anesthesia and surgery. Previous studies have suggested that volatile anesthetics, for example isoflurane, may contribute to such impairment. In the present study, the effects of emulsified isoflurane (EI) exposure on cognitive function, as well as the potential mechanisms, were investigated in animal models. Eight-month-old male rats were administered a single intravenous injection of 8% EI. The rats were then subjected to the Morris water maze test to assess their cognitive functions at different time-points following drug administration. Samples were taken in order to detect the plasma corticosterone concentration and the levels of hippocampal brain-derived neurotrophic factor (BDNF) and nerve growth factor (NGF), as well as the expression of BDNF and NGF in the hippocampal region. The results showed that a single injection of EI caused reversible learning and memory dysfunction in adult rats. It was found that downregulation of BDNF expression may contribute to the isoflurane-induced cognitive impairment of these rats. Increased expression of NGF may be associated with the protection mechanism subsequent to learning and memory function decline, and therefore may accelerate the recovery of cognitive function.

## Introduction

Post-operative cognitive dysfunction (POCD), a severe central nervous system complication, is an acute cognitive deficit following anesthesia and surgery (1,2). POCD may be self-limiting in the majority of patients; however, it may affect the prognosis and life quality of certain individuals (3,4). Therefore, reducing the incidence of POCD is of social importance (5).

brain-derived neurotrophic factor number of studies have suggested that there is a strong link between volatile anesthetics, for example isoflurane, and cognitive impairment (6–8). Emulsified isoflurane (EI) is a novel type of anesthetic that subverts the requirement for specific ventilatory circuits, induces rapid anesthesia and is less environmentally polluting than inhaled isofluorane (9,10). However, very little is known about the effects of EI on the cognitive function of adult rats. Therefore, in the present study the EI-induced alterations and possible mechanisms were investigated.

In the present study, the Morris water maze was used to test spatial learning and memory. Enzyme-linked immunosorbent assays (ELISAs) were performed to measure the levels of plasma corticosterone, brain-derived neurotrophic factor (BDNF) and nerve growth factor (NGF), whilst immunohistochemistry was used to measure BDNF and NGF expression in the hippocampus.

## Materials and methods

### Experimental approval

The animal protocol was approved by the institutional Animal Care and Use Committee of Zunyi Medical College (Zunyi, China). All animal experiments were performed in accordance with the National Institutes of Health Guide for the Care and Use of Laboratory Animals (11).

### Animal groups and anesthetic exposure

Eight-month-old adult male Sprague Dawley (SD) rats, weighing between 250 and 300 g, were obtained from the Laboratory Animal Center of the Third Military Medical University (Chongqing, China). The rats were randomly divided into six groups (12 rats in each group): a control group, a 30% intralipid group (group E) and four EI groups (2 h, 1 day, 7 days, 14 days following recovery from the anesthesia induced by intravenous EI injection; 2h, 1d, 7d and 14d groups, respectively). Rats in the control group did not receive any injection, whilst animals in the 30% intralipid group (group E) received a single intravenous injection of 1.5 ml/kg 30% intralipid (Xian Pharmaceutical, Ltd., Xian, China) via the vena caudalis. Animals in the EI groups were given a single injection of 1.5 ml/kg 8% EI. As previously described (12), the loss of the tail-clamped response and righting reflex were used as the criteria for the anesthesia taking effect, whilst the recovery of the righting reflex was used as the criteria for anesthesia recovery. This method of EI application has been used in previous studies (13,14). An 8% EI (V/V) solution was provided by the New Drug Research Center of Sichuan University (Chengdu, China). Briefly, 1.6 ml liquid isoflurane and 18.4 ml 30% intralipid were mixed in a 20 ml glass ampoule. The EI ampoule was opened immediately prior to use, and any residual drug was discarded.

### Morris water maze

**Figure 1:**
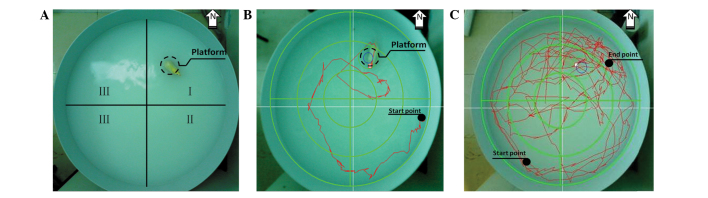
Morris water maze. (A) The water maze consisted of a circular pool and a round platform. The pool was divided into four quadrants. The animals were subjected to (B) the place navigation test and the (C) spatial probe test.

Rats in the EI groups were all tested using the Morris water maze (Chengdu Taimeng Technology Ltd., Chengdu, China) equipped with WMT-100 maze video tracking system (Chengdu Taimeng Software, Chengdu, China) at 2 h, 1 day, 7 days and 14 days following EI injection, respectively. Rats in the 30% intralipid group were subjected to water maze testing 2 h following drug injection together with the control group. The water maze (Fig. 1A) consisted of a circular pool (120 cm in diameter and 60 cm high) and a round platform (15 cm in diameter and 30cm high). The pool was divided into four quadrants: north (where the platform was located), south, east and west. Water in the pool was colored opaque with milk powder (Full Cream Milk Powder; Nestle Shuangcheng Ltd., China) prior to each test to avoid visual cues for the rats.

The test was performed as previously described (15,16) with minor modifications. Each animal was subjected to two tests: a place navigation test (Fig. 1B) and a spatial probe test (Fig. 1C). In the place navigation test, animals were encouraged to find the hidden platform. At the beginning of each trial, the rats were placed into the water facing the wall of the pool in one of the four quadrants. Each rat was allowed 120 sec to find and mount the platform. The amount of time spent finding and mounting the platform (escape latency) and total swimming distance (path length) were calculated using the digital tracking system. If the rats failed to find and mount the platform within 120 sec, the escape latency was recorded as 120 sec. The mean value of the results from four quadrant starting points from 12 rats in each group was used as the final result for the group. The spatial memory of the rats was then analyzed using the spatial probe test. The platform was removed from the pool and the starting point was randomly selected. The swimming time in the former platform quadrant, the percentage of swimming distance in the target quadrant, the average swimming speed and the former platform location passing times within 120 sec were recorded.

### Brain tissue and blood sampling

Immediately following the Morris water maze behavioral tests, rats were anesthetized with 4 mg/100 g 0.1% sodium pentobarbital (Tianjin Damao Chemical Reagent Factory, Tianjin, China) via intraperitoneal injection. Blood (2–4 ml) was collected from the eye orbit of each rat and centrifuged at 300 × g at 6°C for 15 min (Multifuge X1R; Thermo Fisher Scientific, Waltham, MA, USA). Plasma was then collected in order to analyze the corticosterone content by ELISA. Rats were then sacrificed following blood sample collection. Six rats in each group were randomly selected and hippocampi were dissected out and homogenized (T10 basic Ultra-Turrax; IKA, Staufen, Germany). The homogenates were then centrifuged at 900×g (0–4°C) for 15 min. The supernatant was collected and an ELISA was used to measure the expression of BDNF and NGF. The thoracic cavities of the remaining six rats in each group were opened and their aortas were cannulated. The animals were firstly perfused with 200 ml normal saline, then with 300 ml 4% paraformaldehyde (Tianjin Damao Chemical Reagent Factory) until the extremities were rigid. The brains were then removed from the cranial cavity and the tissues were embedded in paraffin. Coronal sections (3 μm thick) were prepared using a freezing microtome (Leica RM 223; Leica Instruments, Nussloch, Germany). A total of 24 sections were obtained from each group, 12 of which were used to determine the expression of BDNF and 12 of which were used for analysis of the expression of NGF by immunohistochemistry.

### Analysis of plasma corticosterone, BDNF and NGF expression using ELISA

Plasma corticosterone, hippocampal BDNF and NGF levels were measured using corticosterone, BDNF and NGF ELISA kits (R&D Systems, Minneapolis, MN, USA) in accordance with the manufacturer’s instructions. Samples were immediately extracted using the methods described above. Briefly, a double-antibody sandwich ELISA was performed. The amount of plasma corticosterone, BDNF and NGF was determined by measuring the absorbance at 450 nm (ELx800; BioTek Instruments, Inc., Winooski, VT, USA). The optical density values from the samples were then used to calculate the concentration based on the standard curve.

### Analysis of BDNF and NGF expression using immunohistochemistry

Immunohistochemistry was conducted as previously described (18–20) with minor modifications. Sections were incubated overnight at 4°C with mouse anti-BDNF antibody immunoglobulin G (IgG; 1:200; Beijing Bioss Biotechnology, Beijing, China) or with mouse anti-NGF antibody IgG (1:100; Beijing Bioss Biotechnology), followed by incubation with biotinylated mouse secondary antibody (Wuhan Boster Biological Technology, Ltd., Wuhan, China). The secondary antibody was amplified using an streptavidin-biotin complex (SABC) kit (Wuhan Boster Biological Technology, Ltd.). The complexes were then visualized using 0.03% diaminobenzidine (Wuhan Boster Biological Technology, Ltd.), and the sections were mounted onto gelatin-coated slides. The slides were air dried overnight at room temperature. Coverslips were mounted using Permount™ Mounting Medium (Zhongshan Golden Bridge Biotechnology Co., Ltd., Beijing, China). The area in the selected region of the hippocampus was measured using Image-Pro Plus software (Leica CW 4000; Leica Instruments). The mean density (MD) of BDNF-positive and NGF-positive cells in the hippocampi was counted. Four distinct views were chosen from each region of the hippocampal CA1, CA2, CA3 and DG regions, and the mean value from the four views was used as the result for the corresponding region. The mean value from 12 sections from each group was used as the final result for that group.

### Statistical analysis

The data in the present study, including the results from the physiological tests, behavioral tests, ELISA and immunohistochemistry, were parametric. They are presented as the mean ± standard deviation. Data from the different groups of animals were analyzed using a one way analysis of variance followed by the Student-Newman-Keuls test following confirmation of normal distribution of the data using SPSS software version 17.0 (SPSS Inc., Chicago, IL, USA). One way repeated measures analysis of variance was used for the comparisons of the values from the same animals at different time-points. P<0.05 was considered to indicate a statistically significant difference.

## Results

### Effect of a single injection of EI on the cognitive function of rats

**Figure 2:**
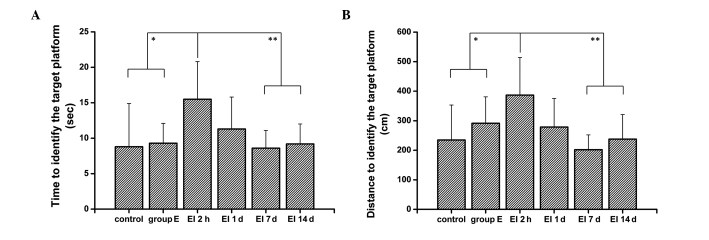
Effect of EI injection on place navigation test performance. (A) Rats in the 2h group took longer to find the platform compared with the rats in the control group, group E, and the 7d and 14d groups. (B) Rats in the 2h group also had a longer path length to find the platform. Data are presented as the mean ± standard deviation; n=12. ^*^P<0.01; ^**^P<0.05. EI, emulsified isoflurane; E, 30% intralipid.

The adult SD rats started to manifest restlessness following the EI injection (0.1–0.2 ml) and all the rats were clearly anesthetized. The anesthesia recovery time of the SD rats was 46.5±12.3 sec (P<0.05) in the present study. As shown in Fig. 2, in the place navigation test, the rats in the 2h group spent significantly more time and had a longer path length to find the platform compared with rats in the control and the E groups (P<0.01). These results suggest that the rats developed cognitive dysfunction following the EI injection. There was also a significant increase in the escape latency and path length of the 2h group compared with the 1d, 7d and 14 groups (P<0.05). However, there was no significant difference in the escape latency and path length among the control, the E, and the 1d, 7d and 14 groups (P>0.05). Therefore, these results suggest that the EI-induced cognitive dysfunction may be reversed in a relatively short period of time and will not cause long-term damage to the cognitive function of animals.

**Figure 3:**
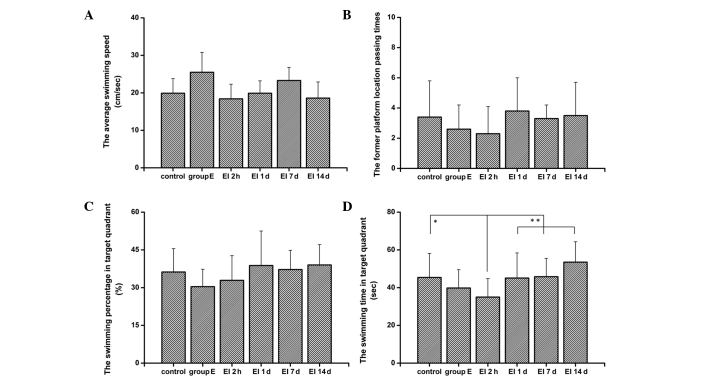
Effect of EI injection on spatial probe test performance. (A) Average swimming speed, (B) platform passing times, and (C) platform quadrant swimming times all showed no significant differences between the groups. (D) Rats in the 2h group spent less time in the target quadrant compared with the control, 1d, 7d and 14d groups. Data are presented as the mean ± standard deviation; n=12. ^*^P<0.01; ^**^P<0.05. EI, emulsified isoflurane; E, 30% intralipid.

The results from the spatial probe test (Fig. 3) revealed that there were no significant differences between rats in each test group in the platform quadrant swimming time, the average swimming speed and the platform passing times. Similar to the results from the place navigation test, rats in the 2h group spent less time in the target quadrant than the control group and the 1d, 7d and 14d groups did (P<0.01, compared with control group; P<0.05 compared with the 1d, 7d and 14 groups).

### Cognitive dysfunction may not be due to plasma corticosterone levels

**Figure 4:**
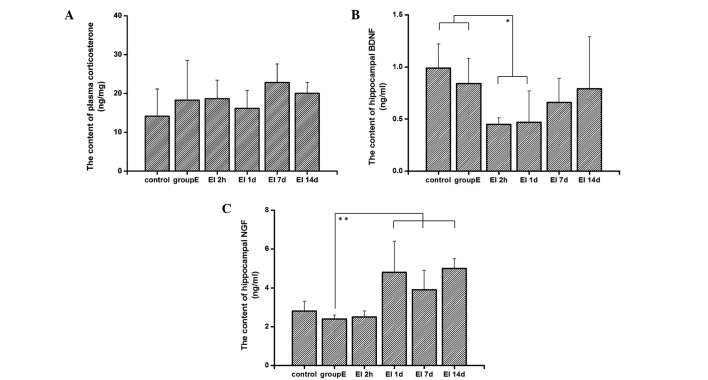
Changes in plasma corticosterone and hippocampal BDNF and NGF expression levels analyzed using an ELISA. (A) No significant differences in plasma corticosterone levels were observed between groups. (B) There was an increase of BDNF expression in the 7d and 14d groups compared with the 2h group and the 1d group. (C) The levels of NGF in the 1d, 7d and 14d groups all showed an increase compared with that in the control group, group E and the 2h group. Data are presented as the mean ± standard deviation; n=12. ^*^P<0.01; ^**^P<0.05. BDNF, brain-derived neurotrophic factor; NGF, nerve growth factor; ELISA, enzyme-linked immunosorbent assay; EI, emulsified isoflurane; E, 30% intralipid.

The results from the present study showed that rats in the control group had lower levels of plasma corticosterone compared with the other groups. This may be due to the stress reaction as a result of the drug injection. However, there was no significant difference between the plasma corticosterone levels of rats in different groups (Fig. 4A). This suggests that plasma corticosterone levels were not affected by EI anesthesia, indicating that corticosterone levels do not have a role in EI-induced cognitive dysfunction.

### Levels of BDNF were decreased in the hippocampi of rats

As shown in Fig. 4B, the BDNF levels in the hippocampi of rats in the 2h and 1d groups were significantly lower compared with those of the control group (P<0.05). There were increases of BDNF levels in the 7d and 14d groups compared with those in the 2h and in the 1d group. The BDNF levels in the 7d and 14d groups showed no difference from those in the control and group E (P>0.05). These results suggest that EI injection causes a reduction of BDNF expression in the hippocampus, which may be responsible for the cognitive impairment observed in SD rats following EI anesthesia. The lowest levels of BDNF expression were observed 2 h following recovery from anesthesia; however, the BDNF levels then returned to previous levels.

### Expression levels of NGF were increased in the hippocampi of rats

As shown in Fig. 4C, the levels of NGF were increased 1 day following recovery from the EI injection and the increase was sustained; the levels of NGF in groups 1d, 7d and 14d all showed an increase compared with those in the control, E and 2h groups (P<0.05). These results indicate that NGF, as a nerve protective factor, starts to restore nerve functions following EI injection and possibly attenuates the reduction of BDNF expression, consequently promoting the recovery progress of EI-induced cognitive impairment.

### Effect of EI on the expression of BDNF and NGF detected using immunohistochemistry

**Figure 5:**
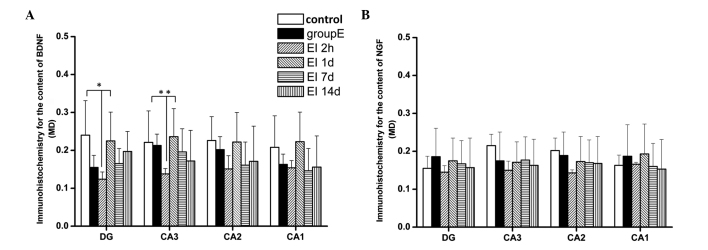
Effect of EI on the levels of BDNF and NGF, detected using immunohistochemistry. (A) The MD of BDNF-positive cells in the DG and CA3 region the 2 h group was lower compared with those in the control and 1d groups. (B) There were no differences with regard to NGF expression in different hippocampal regions. Data are given as means ± SD; n=12. ^*^P<0.01; ^**^P<0.05. EI, emulsified isoflurane; BDNF, brain-derived neurotrophic factor; NGF, nerve growth factor MD, mean density.

**Figure 6:**
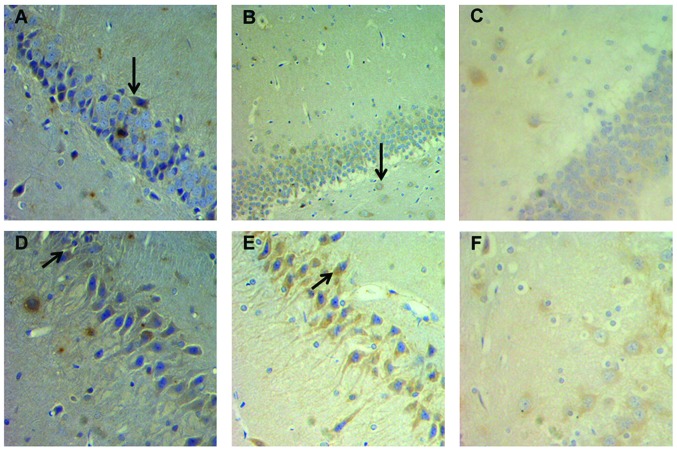
Representative images demonstrating changes in BDNF and NGF expression. The MD of BDNF-positive cells in the DG region in the (C) 2 h group was lower compared with those in (A) the control group and (B) the 1d group. The MD of BDNF-positive cells in the CA3 region of (F) the 2h group was also lower compared with those in (D) the control group and (E) the 1d group. BDNF, brain-derived neurotrophic factor; NGF, nerve growth factor MD, mean density. The arrows indicate the BDNF positive cells.

The results from the immunohistochemical analysis revealed (Fig. 5 and 6) that the MDs of BDNF-positive cells in the DG and CA3 region in the 2h group were significantly lower compared with those in the control and 1d groups (P<0.05). There were no significant differences in BDNF expression in the CA2 and CA1 regions among the groups (P>0.05). There was also no significant difference between the BDNF levels in different brain areas in the 1d, 7d and 14d groups (P>0.05). Similar to the results observed from the ELISA test, EI exposure markedly decreased the levels of BDNF in the hippocampal DG and CA3 region 2 h following EI anesthesia recovery. There were no significant differences among all experimental groups with regards to NGF expression in the different hippocampal regions (P>0.05).

## Discussion

The Morris water maze is a test developed by the British psychologist Richard G. Morris in the 1980s to assess spatial memory and learning of rodents, which has become one of the ‘gold standards’ of behavioral neuroscience (20). The Morris water maze usually consists of two parts: the place navigation test and the spatial probe test. The place navigation test reflects the spatial learning ability of animals; escape latency and path length have been shown to be negatively correlated with spatial learning ability. The spatial probe test is used to determine the spatial association and reference memory. Swimming time in the former platform quadrant, the percentage of swimming distance in target quadrant, the average swimming speed and the former platform location passing times within 120 sec are positively correlated with reference memory ability (21).

The results from this experiment in the present study demonstrated that EI exposure may cause transient deficits in water maze performance. Compared with those in the control group and the E group, the escape latency and path length to the target quadrant was prolonged 2 h after the EI injection. In addition, the time that rats were in the quadrant where the platform had been located was shortened. These results indicate that the learning and memory ability decline may be due to the effect of the EI injection. However, 1 day following EI injection, the Morris water maze results showed no significant difference compared with those in the E and the control groups. Several previous studies have shown impairments of learning and memory following exposure to isoflurane inhalation anesthesia and also found that these isoflurane-induced cognitive deficits are reversible (22–25). The results from the present study are in accordance with these previous studies and they suggest that the cognitive function of rats may completely recover to pre-anesthesia levels. It was shown in the present study that EI-induced cognitive impairment is not a persistent and irreversible process. No difference was observed between the results from the Morris water maze from the 7d and 14d groups compared with the control and E groups, suggesting that after two weeks of recovery, the cognitive function of rats has already recovered to a stable stage.

The central nervous system mainly contains mineralocorticoid receptors and glucocorticoid receptors (GRs). The hippocampus is a brain area crucial for memory storage and is very vulnerable to the effects of glucocorticoids due to its high levels of GRs. The high levels of corticosterone generated as a stress response bind to GRs. The activated GRs then induce the downregulation of nerve survival genes and the upregulation of nerve cell apoptosis genes, therefore leading to the reduction of hippocampal nerve cell synapses and the induction of the apoptosis process of hippocampal nerve cells and cognitive dysfunction (26). Previous studies have shown that external or internal corticosterone reduces BDNF expression. Exogenous dexamethasone downregulates the expression of the tyrosine kinase receptor, reduces phospholipase and Ca^2+^ channel activation and causes learning and memory dysfunction (27,28). However, the results from the present study demonstrate that there were no significant differences in plasma corticosterone levels among all groups (P>0.05), suggesting that the EI-induced cognitive dysfunction was not due to an increase in plasma corticosterone levels.

BDNF and NGF are important in the development, survival and maintenance of neurons in the central nervous system (29). The hippocampus has a key role in memory and spatial location since it is an important area of the brain required for learning and memory. Previous studies have demonstrated that the downregulation of BDNF and NGF in the brain may result in memory and learning deficits (30,31).

The results from the present study showed that the MD of BDNF-positive cells decreased in the DG and CA3 regions of the hippocampus 2 h following EI injection, which is in accordance with the results obtained from the ELISA, indicating that the reduction in BDNF caused by anesthesia toxicity is a cause of the EI-induced cognitive dysfunction. The results also demonstrated that there was a marked increase and recovery of BDNF expression levels from 1 day following anesthesia, which eventually reached normal levels. There was no significant difference observed between BDNF concentrations in the 7d and 14d groups compared with those in the control and E groups, indicating that the EI-induced cognitive dysfunction was reversible.

In the present study, the effect of emulsified isoflurane on the cognitive function of rats was investigated for the first time, to the best of our knowledge. It was found that a single injection of EI caused transient cognitive impairment in rats, which may be due to the downregulation BDNF expression, which is similar to the results observed with isoflurane. However, the effect of repeated intravenous EI administration on cognitive function requires further investigation.
